# A time-series minimally invasive transverse aortic constriction mouse model for pressure overload-induced cardiac remodeling and heart failure

**DOI:** 10.3389/fcvm.2023.1110032

**Published:** 2023-02-20

**Authors:** Xia Wang, Xinxin Zhu, Li Shi, Jingjing Wang, Qing Xu, Baoqi Yu, Aijuan Qu

**Affiliations:** ^1^Beijing Key Laboratory for HIV/AIDS Research, Center for Infectious Diseases, Beijing Youan Hospital, Capital Medical University, Beijing, China; ^2^Department of Physiology and Pathophysiology, School of Basic Medical Sciences, Capital Medical University, Beijing, China; ^3^Key Laboratory of Remodeling-Related Cardiovascular Diseases, Ministry of Education, Beijing Key Laboratory of Metabolic Disorder-Related Cardiovascular Diseases, Beijing, China; ^4^Laboratory of Animal Facility, Capital Medical University, Beijing, China; ^5^Core Facility Centre, Capital Medical University, Beijing, China

**Keywords:** heart failure, cardiac remodeling, cardiac hypertrophy, cardiac fibrosis, transverse aortic constriction

## Abstract

Transverse aortic constriction (TAC) is a widely-used animal model for pressure overload-induced cardiac hypertrophy and heart failure (HF). The severity of TAC-induced adverse cardiac remodeling is correlated to the degree and duration of aorta constriction. Most studies of TAC are performed with a 27-gauge needle, which is easy to cause a tremendous left ventricular overload and leads to a rapid HF, but it is accompanied by higher mortality attributed to tighter aortic arch constriction. However, a few studies are focusing on the phenotypes of TAC applied with a 25-gauge needle, which produces a mild overload to induce cardiac remodeling and has low post-operation mortality. Furthermore, the specific timeline of HF induced by TAC applied with a 25-gauge needle in C57BL/6 J mice remains unclear. In this study, C57BL/6 J mice were randomly subjected to TAC with a 25-gauge needle or sham surgery. Echocardiography, gross morphology, and histopathology were applied to evaluate time-series phenotypes in the heart after 2, 4, 6, 8, and 12 weeks. The survival rate of mice after TAC was more than 98%. All mice subjected to TAC maintained compensated cardiac remodeling during the first two weeks and began to exhibit heart failure characteristics after 4 weeks upon TAC. At 8 weeks post-TAC, the mice showed severe cardiac dysfunction, hypertrophy, and cardiac fibrosis compared to sham mice. Moreover, the mice raised a severe dilated HF at 12 weeks. This study provides an optimized method of the mild overload TAC-induced cardiac remodeling from the compensatory period to decompensatory HF in C57BL/6 J mice.

## Introduction

Heart failure (HF) is a consequence of various cardiovascular diseases and is still one of the leading causes of mortality worldwide, which remains a major clinical and public health problem (1–3). The development of HF is characterized by a process of adverse cardiac remodeling (4), and it is mainly attributed to increased pressure overload such as hypertension (5, 6). Therefore, it is important to uncover the cellular and molecular mechanisms of HF for better understanding and new therapeutic targets.

Transverse aortic constriction (TAC) has been the preferred murine model of adverse cardiac remodeling induced by pressure overload, which plays an important role in preclinical studies (7–11) since it was first built by Rockman et al. (12). The severity of adverse cardiac remodeling induced by TAC largely depends on the degree of aorta constriction and the duration of constriction. Therefore, there is considerable variability in TAC during the cardiac remodeling progression to an overt HF (13). At present, several minimal invasiveness TAC models with low mortality have been developed (9–11, 14–17), which has made the TAC model more effective and accurate. The mice subjected to TAC go through cardiac hypertrophy, cardiac fibrosis, and inflammation, and eventually develop cardiac dilation and HF. The progression of cardiac remodeling and HF induced by TAC relies on the degree and duration of constriction of the aorta (13). Although increasing the degree of aortic constriction, frequently using a 27-gauge needle, can increase the severity of pressure response in the left ventricular wall and increase the probability of more animal transition from compensated cardiac hypertrophy to HF, the mortality rate is higher than expected (7, 8, 18).

The TAC conducted with a 25-gauge needle presents a mild cardiac remodeling progression with lower mortality (19). Therefore, a 25-gauge needle can cause a much milder constriction of the aorta arch and could be very useful for studying hypertensive cardiac remodeling in mice, or studies with more susceptibility to mortality. In addition to the severity of constriction, its duration is also responsible for the variation of TAC response. Moreover, few studies have focused on time-series phenotypes of TAC models induced by different durations of aortic constriction (20). In C57BL/6 J mice, the potential time-series of adverse cardiac remodeling induced by TAC applied with a 25-gauge needle at different durations of constriction also remains unknown. Although C57BL/6 J mice are one of the most used strains, their susceptibility to HF development is also controversial (20, 21). Therefore, generating a reproducible murine cardiac remodeling model is instrumental for investigating the mechanisms of HF progress.

In this study, a simple and less invasive TAC method was performed. The procedure involves the fewest apparatuses and less damage to the mice. This TAC method neither requires cutting of the ribs and intercostal muscles nor tracheal intubation with a ventilation setup. Moreover, TAC surgery with a 25-gauge needle has a greater survival rate, above 98%, and its operation time is only 10–15 min. Then, time-series cardiac remodeling phenotypes induced by a much milder TAC applied with a 25-gauge needle were analyzed in C57BL/6 J mice. The phenotype results indicate that HF and cardiac dilation raised in 8 weeks or later in TAC mice. This study provides a deep insight into a 25-gauge TAC-induced cardiac remodeling from the compensatory period to the decompensatory stage and leads to HF in C57BL/6 J mice.

## Materials and methods

### Animals

C57BL/6 J mice were purchased from Charles River Company (Beijing, China). All mice were kept under specific pathogen-free conditions, a standard 12-h light/dark cycle in individually ventilated cages, and free access to a normal chow diet and water. All experimental procedures were in accordance with the U.S. National Institutes of Health Guidelines for the Care and Use of Laboratory Animals. All animal studies were approved under the project license AEEI-2018-127 granted by the ethics board of Capital Medical University.

### Minimally invasive transverse aortic constriction surgery

A minimally invasive transverse aortic constriction (TAC) method without standard chest opening has been established (17). In this study, the 10~12-week-old male C57BL/6 J mice (23~27 g) were randomized to be subjected to TAC or sham surgery. All mice were anesthetized with a single intraperitoneal injection of supersaturated 2,2,2-tribromoethanol (T48402, Sigma-Aldrich, St. Louis, MO, United States) saline solution at a dose of 10~13 μL g^−1^ and fixed on an operation plate. Adequate sedation was determined by the lack of toe-pinch reflex. A topical depilatory agent was applied to the neck and chest, and the area was cleaned with 75% alcohol.

**Figure 1:**
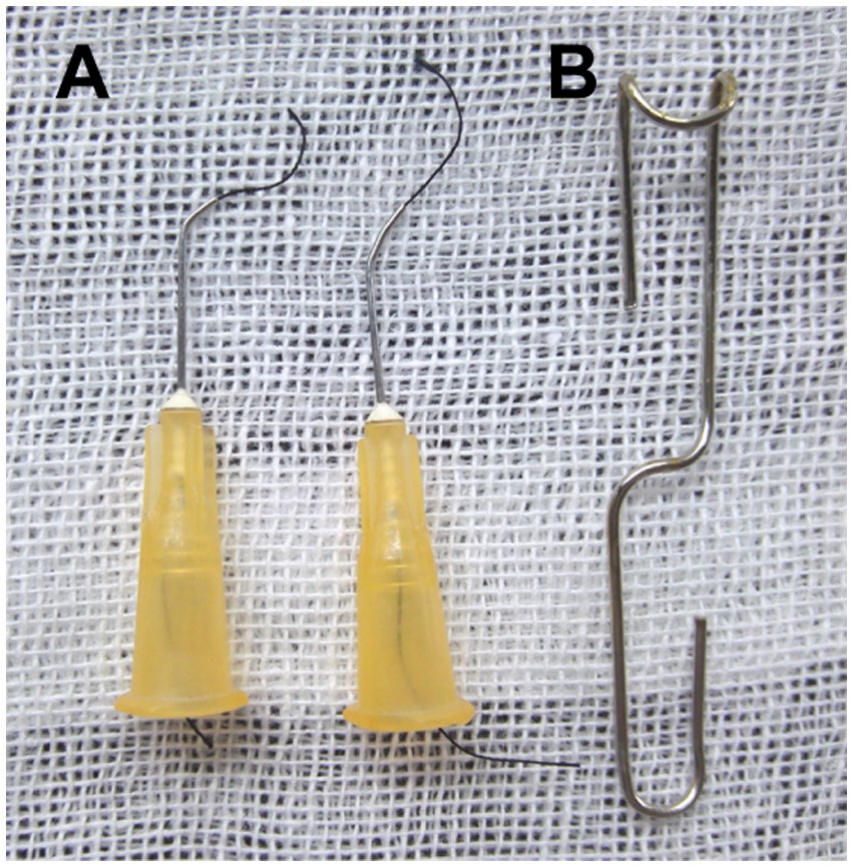
Homemade surgical instruments. **(A)** Creating the ligation spacer and curved needle with suture: bend the blunt 25-gauge needle 30°~45° with a needle holder. **(B)** Making a simple surgical retractor with the paper clip, as illustrated previously.

**Figure 2:**
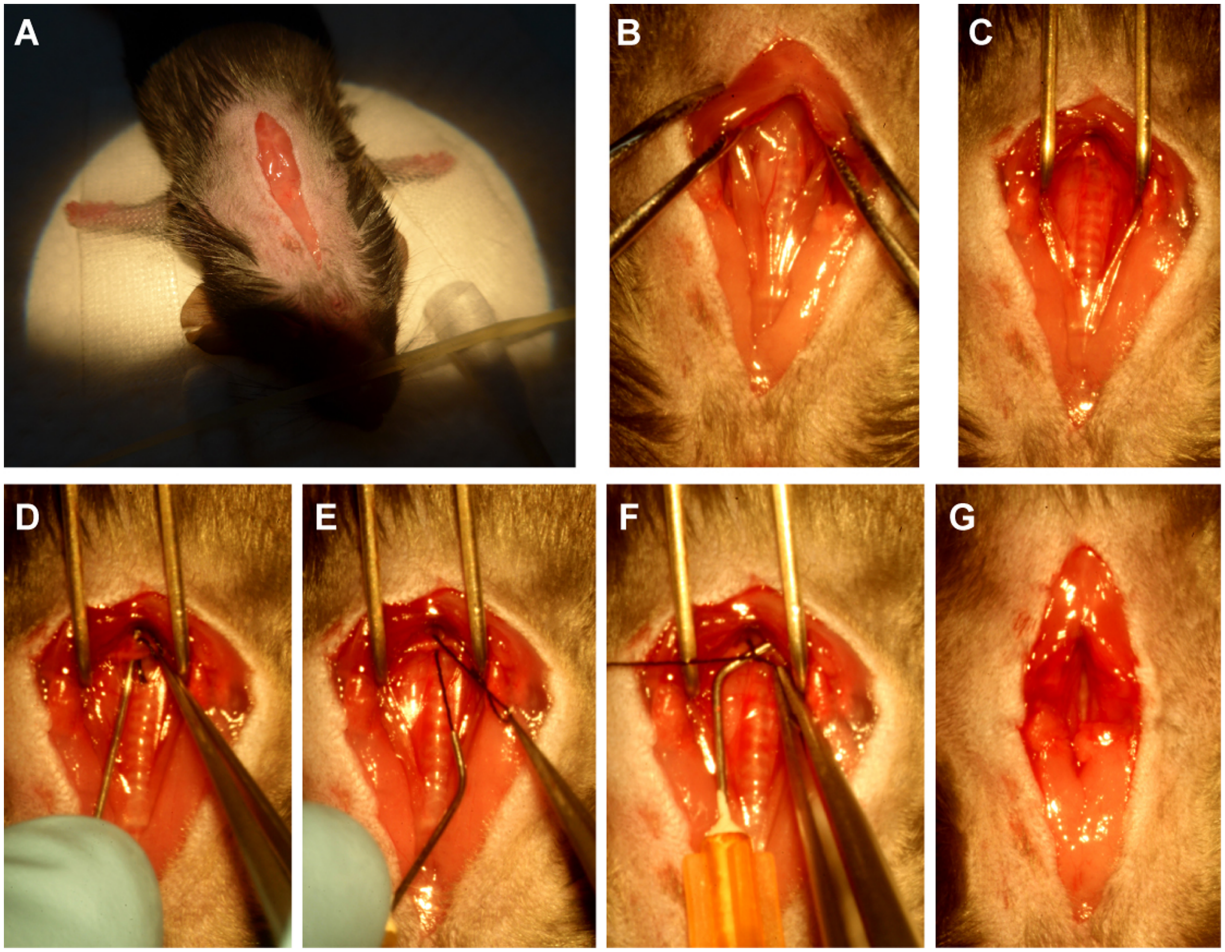
The procedure of minimally invasive TAC. **(A)** Open a 1.5 ~ 2 cm incision at the midline of the neck and chest. **(B)** Cut the sternum stem about 3~5 mm. **(C)** Separate the aortic arch from the nearby connective tissue by using the above-mentioned simple surgical retractor. **(D)** Guide the suture under the aortic arch by using the above-mentioned curved needle with suture. **(E)** Pull out of the suture from the curved needle. **(F)** Binding a constriction of the desired diameter with the above-mentioned 25-gauge curved needle. **(G)** Completed constriction.

Before the surgery, several 25-gauge blunt needles and a simple retractor made of paper clip were prepared (Figure 1). Under a dissecting microscope (Model SZ2-ILST, OLYMPUS Corporation, TOKYO, Japan), the mouse was placed in the supine position, the skin was opened, and a 1.5~2 cm incision at the midline of the neck and chest was made (Figure 2A). The thyroid gland was pulled toward the head by gently separating connective tissues. Then, the sternocleidomastoid muscle layer on the trachea was separated toward both sides. Next, the sternum stem was cut along the midline and slightly separated (Figure 2B), and the thymus covering the aortic arch was separated from it. Finally, the aortic arch and two carotid arteries were fully exposed by the simple retractor (Figure 2C).

A 25-gauge curved needle with a 7-0 suture, as shown in Figure 1A, was placed under the arch and pushed to perforate between the vessel wall and connective tissue on the other side (Figure 2D). Then, the suture was pulled out of the curved needle (Figure 2E), and it was immediately taken out from the aortic arch and placed above it as a spacer for ligation (Figure 2F). Finally, the knotted position was retracted successfully, and the spacer was gently withdrawn (Figure 2G). When the needle was removed, the diameter of the aortic arch was narrowed to 0.5 mm. The end of the suture was cut, and the skin was closed with a 4–0 suture in a continuous suture pattern. After surgery, all mice were allowed to recover on a warming pad until they were fully awake. The sham surgery was subjected to an identical operation in which the aortic arch was visualized but not banded.

All TAC and sham mice were randomly divided into five groups for the experiment: 2 weeks group (2 W), 4 weeks group (4 W), 6 weeks group (6 W), 8 weeks group (8 W), and 12 weeks group (12 W). All groups were observed after 1 week of surgery.

### Echocardiography

After 3~7 days of surgery, aortic flow peak velocity (AV Peak Vel) at the TAC constriction band site was measured using color and pulsed-wave Doppler (Vevo2100; VisualSonics, Inc., Toronto, ON, Canada). Briefly, mice were shaved and anesthetized with isoflurane (2%–4% for induction and 1%–1.5% for maintenance) and were placed in the supine position on a heated platform with ECG electrodes attached to monitor the heart rate (>550 bpm). The pulsed-wave Doppler was used to measure blood speed in either TAC (total 50) or sham (total 25) mice, and the peak pressure was also calculated.

Cardiac function was evaluated at a time-series by echocardiography using a high-resolution small animal imaging system (Vevo 2100; VisualSonics, Toronto, ON, Canada) as described (22, 23). B-Mode and M-Mode of parasternal long and short axis were measured at the level of the papillary muscles (Sham 2 W group, *n* = 22; TAC 2 W group, *n* = 34; Sham 4 W group, *n* = 16; TAC 4 W group, *n* = 22; Sham 6 W group, *n* = 18; TAC 6 W group, *n* = 19; Sham 8 W group, *n* = 19; TAC 8 W group, *n* = 26; Sham 12 W group, *n* = 12; TAC 12 W group, *n* = 24). By using Vevo LAB 2.1.0 software, the following parameters were measured digitally from the M-mode tracings as follows: heart rate, diastolic and systolic left ventricular anterior wall, diastolic and systolic left ventricular internal dimensions, and diastolic and systolic left ventricular posterior wall. Based on these measurements, diastolic and systolic left ventricular volume, left ventricular mass, left ventricular ejection fraction (EF%), and left ventricular fractional shortening (FS%) were also calculated.

### Morphological analyses

Mice were euthanized at 2, 4, 6, 8, and 12 W after operation, respectively. Hearts were fixed in 10% phosphate-buffered formalin, embedded in paraffin, and sectioned (4 μm). Hematoxylin and eosin (H&E), Masson’s trichrome, and wheat germ agglutinin (WGA) staining were performed on the sections using standard procedures as previously described (23). Images were obtained using a high-capacity digital slide scanner (Pannoramic SCAN, 3DHISTECH, Budapest, HUN). The fibrotic area (collagen area/total area) was dyed by Masson’s trichrome staining (Sham 2 W group, *n* = 10; TAC 2 W group, *n* = 15; Sham 4 W group, *n* = 8; TAC 4 W group, *n* = 8; Sham 6 W group, *n* = 8; TAC 6 W group, *n* = 8; Sham 8 W group, *n* = 10; TAC 8 W group, *n* = 15; Sham 12 W group, *n* = 8; TAC 12 W group, *n* = 8) and cross-sectional area of the cardiomyocytes (Sham 2 W group, *n* = 10; TAC 2 W group, *n* = 10; Sham 4 W, *n* = 10; TAC 4 W group, *n* = 14; Sham 6 W group, *n* = 8; TAC 6 W group, *n* = 11; Sham 8 W group, *n* = 14; TAC 8 W group, *n* = 15; Sham 12 W group, *n* = 10; TAC 12 W group, *n* = 12) was determined using Image-Pro Plus 6.0 software (Media Cybernetics, Rockville, MD, United States).

### Quantitative real-time PCR

Total RNA in the heart tissues was extracted by the TRIzol reagent (Invitrogen, New York, United States). The first cDNA strand was synthesized from 2 μg of total RNA using the GoScript™ Reverse Transcription System (Promega, Southampton, United Kingdom). Quantitative real-time (qPCR) was performed using SYBR Green Master Mix (TaKaRa, Tokyo, Japan) with CFX Connect Real-Time System (Bio-Rad, Hercules, CA). Amplification was performed as follows: 95°C for 3 min, 95°C for 30 s, and 60°C for 45 s for each step of 40 cycles. The expression of hypertrophic genes *Anf*, *Bnp*, and *Myh7* and fibrotic genes *Col1a2* and *Col3a1* (Sham 2 W group, *n* = 10; TAC 2 W group, *n* = 20; Sham 4 W, *n* = 8; TAC 4 W group, *n* = 14; Sham 6 W group, *n* = 9; TAC 6 W group, *n* = 15; Sham 8 W group, *n* = 13; TAC 8 W group, *n* = 19; Sham 12 W group, *n* = 10; TAC 12 W group, *n* = 17) were measured using qPCR. mRNA levels were normalized to the level of the endogenous housekeeping gene *Actb* and calculated with the comparative cycle threshold method (ΔΔCT).

Primers sequences are as follows: *Anf* (Forward: CACAGATCTGATGGATTTCAAGA, Reverse: CCTCATCTTCTACCGGCATC); *Bnp* (Forward: 5′-GAAGGTGCTGTCCCAGATGA-3′, Reverse: 5′-CCAGCAGCTGCATCTTGAAT-3′); *Myh7* (Forward: 5′-GATGTTTTTGTGCCCGATGA-3′, Reverse: 5′-CAGTCACCGTCTTGCCATTCT-3′); *Col1a2* (Forward: 5′-AGTCGATGGCTGCTCCAAAA-3′, Reverse: 5′-AGCACCACCAATGTCCAGAG-3′); *Col3a1* (Forward: 5′-TCCTGGTGGTCCTGGTACTG-3′, Reverse: 5′-AGGAGAACCACTGTTGCCTG-3′); *Actb* (Forward: 5′-ATGGAGGGGAATACAGCCC-3′, Reverse: 5′-TTCTTTGCAGCTCCTTCGTT-3′).

### Statistical analysis

All data in the study are expressed as mean ± S.E.M and were calculated and plotted using GraphPad Prism 8.0 software (GraphPad Software, La Jolla, CA). For statistical comparisons, the determination of normal distribution was first evaluated. Then, potentially similar variances were evaluated in normally distributed data. Student’s *t*-test was performed for two group comparisons and ANOVA for the comparison of groups for which similar variance tests were passed. Nonparametric tests were used where data were not normally distributed. Log-rank (Mantel–Cox) test and Gehan–Breslow–Wilcoxon test were performed for the survival rate of mice after TAC 12 W (Sham 12 W group, *n* = 43; TAC 12 W group, *n* = 70). In all cases, significance was attributed to differences for which the two-tailed probability was <0.05.

## Results

### TAC induces significant pressure overload of the heart

Compared to the traditional open-chest TAC, this minimally invasive TAC procedure is simpler and easier to perform, time, and labor-saving. As shown in Figure 1, the preparation of the primary tools for this surgery is simple and minimal, requiring 2~3 bent, 25-gauge blunt needles, and a simple retractor made with a paper clip. Once the thyroid had been retracted, the sternum was cut, and the thymus was moved to fully expose the aortic arch. Subsequent ligation of the aortic arch was very simple and easy (Figure 2).

**Figure 3:**
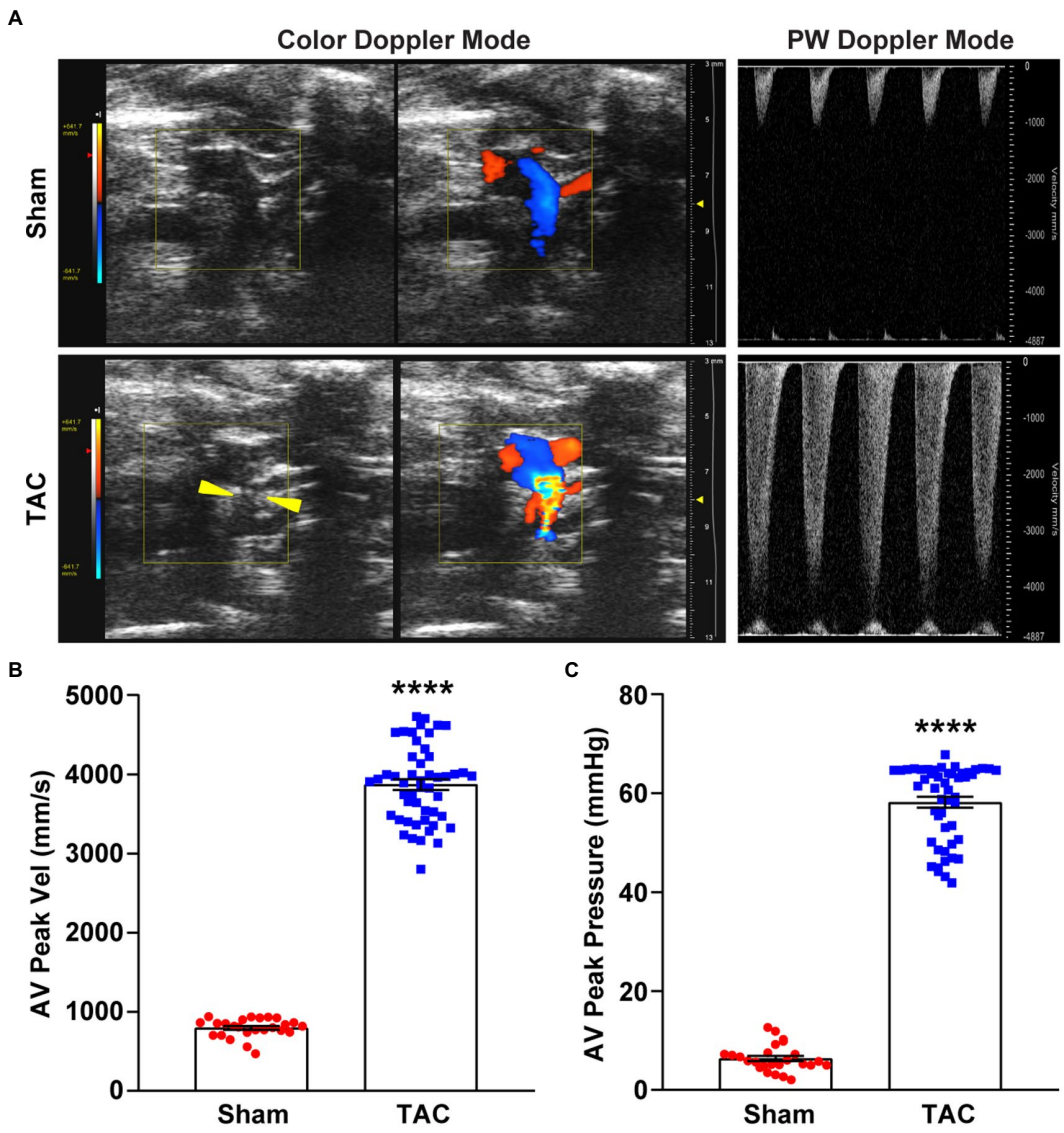
Color and pulsed-wave Doppler ultrasound. **(A)** Representative color Doppler imaging of an aortic flow velocity peak of the non-ligated (sham) mouse (top) and the ligated (TAC) mouse (bottom). The constriction between the two carotid arteries on the aortic arch is visible (yellow arrowheads). **(B)** Quantification of the AV peak velocity after surgery. **(C)** AV peak pressure was calculated by doppler velocities. *n* = 25~50. Unpaired *t*-test for two-group comparisons, ****p < 0.0001 vs. Sham.

Color Doppler and pulsed-wave Doppler were used to verifying the success of aortic arch constriction and determine the flow velocities of the aortic arch (24, 25). However, many studies may not have confirmed the success of aortic contraction (13). In this study, the constricted transverse aorta could be clearly visualized by color Doppler (Figure 3A), 7 days after surgery. Also, by using pulsed-wave Doppler, it was possible to verify that the flow velocity peak was increased at the contraction site. In the banding site of TAC mice, a successful constriction could be clearly observed which was shown by a significant increase in AV flow velocity peak (>2,800 mm/s) compared to a sham mouse with no constriction (Figure 3B). Moreover, the AV pressure peak was used to assess TAC severity. The pressure peak (>40 mmHg) was able to overload the left ventricular wall (Figure 3C). These results show that the TAC applied with a 25-gauge needle successfully produced left ventricular wall pressure overload in C57BL/6 J mice.

### TAC induces time-series cardiac remodeling in mice subjected to surgery with a 25-gauge needle

**Figure 4:**
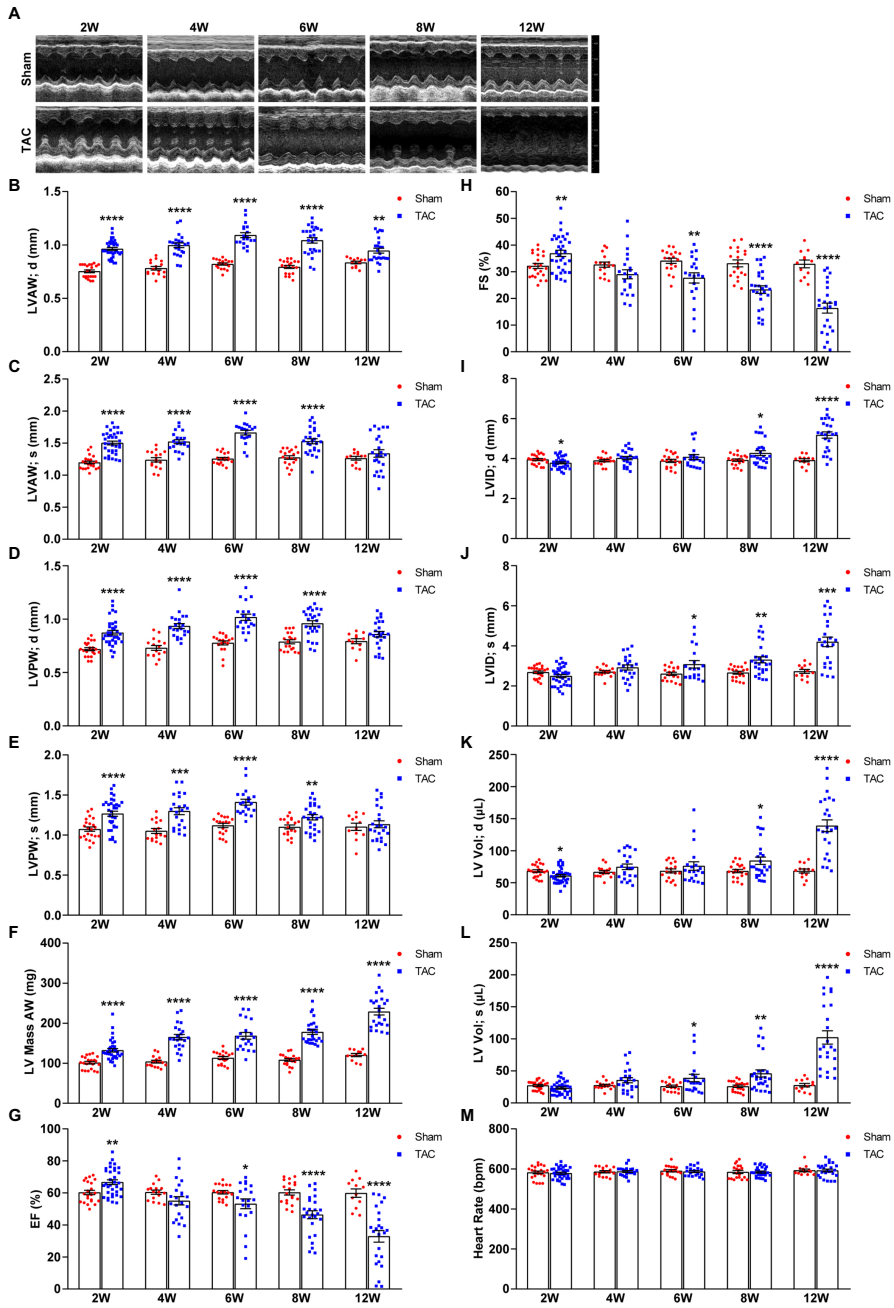
Echocardiography at various time points after surgery. **(A)** Representative two-dimensional guided M-mode echocardiogram of the various groups. **(B)** The thickness of the diastolic left ventricular anterior wall. **(C)** Systolic left ventricular anterior wall. **(D)** Diastolic left ventricular posterior wall. **(E)** Systolic left ventricular posterior wall. **(F)** Left ventricular mass AW. **(G)** Quantification of ejection fraction (EF). **(H)** Quantification of fractional shortening (FS). **(I)** Diastolic left ventricular internal dimensions. **(J)** Systolic left ventricular internal dimensions. **(K)** Diastolic left ventricular volume. **(L)** Systolic left ventricular volume. **(M)** Heart rate. *n* = 12~34. Unpaired *t*-test for two-group comparisons, *p < 0.05, **p < 0.01, ***p < 0.001, ****p < 0.0001 vs. corresponding Sham.

TAC-treated mice develop cardiac hypertrophy, cardiac fibrosis, inflammation, and eventually cardiac dilation and HF. The progression of this adverse cardiac remodeling induced by TAC is associated with the duration of aortic constriction (9, 13). However, very few studies observed a time-series cardiac remodeling phenotype induced by TAC (11, 20, 21), especially in C57BL/6 J mice applied with a 25-gauge needle. In this study, echocardiography was used to evaluate the cardiac morphology and function changes by analyzing the left ventricle wall thickness and systolic functional parameters at 2, 4, 6, 8, and 12 weeks after TAC surgery in C57BL/6 J mice. During echocardiography, all mice were posited on a heated platform with ECG electrodes attached, to monitor the heart rate > 550 bpm. Compared to the sham group, all mice subjected to TAC surgery successfully developed adverse cardiac remodeling (Figure 4A). First, the echocardiography results showed that the left ventricular wall thickness was increased in all TAC groups. Further analysis showed time-series changes in the left ventricular anterior wall (LVAW) among the five TAC groups. Within 6 weeks, the LVAW thickness of TAC mice gradually thickened, but it began to become thinner after 8 weeks, and by the end of 12 weeks the LVAW thickness was even thinner (Figures 4B,C). Similar results were also observed for the thickness of the left ventricular posterior wall (LVPW; Figures 4D,E). Moreover, left ventricular mass (LV mass) was also calculated in the study, which is one of the most important parameters of cardiac remodeling. Results showed that when compared to sham mice, the LV mass of TAC mice increased in a time-series (Figure 4F). Second, compared to the sham group, left ventricular systolic function analysis showed that the ejection fraction (EF) and fractional shortening (FS) increased compensatory within 2 weeks after TAC, but they gradually decreased on week 4, and significantly decreased over 8 weeks, especially at week 12 (Figures 4G,H). Third, the left ventricular internal dimensions (LVID) and left ventricular volume (LV vol), which are important parameters for left ventricular remodeling and cardiac dysfunction, were also analyzed. Compared to sham mice, the diastolic LVID and systolic LVID in TAC mice were decreased at week 2 and then progressively increased within 4~12 weeks after TAC (Figures 4I,J). Similar results for the diastolic LV vol and systolic LV vol (Figures 4K,L) were also observed. During the echocardiography, mice were placed on a heated platform with ECG electrodes attached to monitor the heart rate > 550 bpm, and there was no significant difference among all groups (Figure 4M).

Taken together, C57BL/6 J mice subjected to TAC applied with a 25-gauge needle maintained a compensated state with increased EF and FS in 2 weeks post-surgery and gradually developed to the decompensatory period over 4 weeks after TAC. At 4 weeks, TAC mice were in the early phase of cardiac dysfunction, but they developed serious cardiac dysfunction with extremely low EF and FS and extremely increased cardiac diameters and volumes over 8 weeks post-TAC and even congestion at week 12.

**Figure 5:**
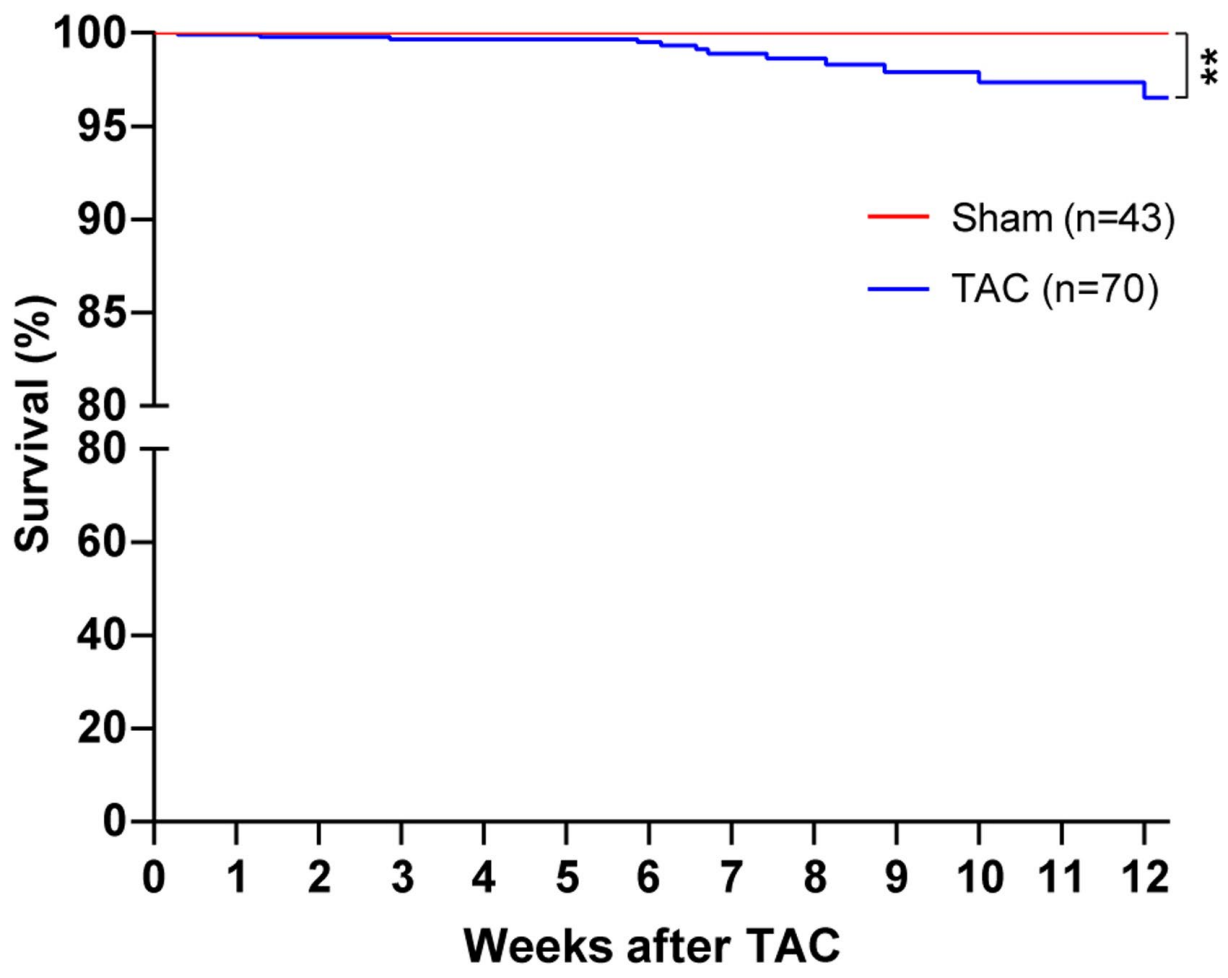
Survival of mice after TAC surgery. C57BL/J mice were randomized to TAC with a 25-gauge needle or sham surgery. The survival of mice was monitored for 12 weeks after surgery. Log-rank (Mantel–Cox) test and Gehan–Breslow–Wilcoxon test were performed for the survival rate of mice after TAC. ^**^p < 0.01 vs. Sham.

In our study, mice survival after TAC surgery was also analyzed for 12 weeks and found that mice subjected to a 25-gauge TAC had >98% survival in the perioperative period (24 h) and 1 week after surgery (Figure 5). Moreover, the survival of 8 weeks and 12 weeks after TAC was 88.57% and 82.86%, respectively. Taken together, this TAC method applied with a 25-gauge needle greatly improves the survival rate after operation in C57BL/6 J mice.

### A 25-gauge needle TAC induces cardiac hypertrophy

**Figure 6:**
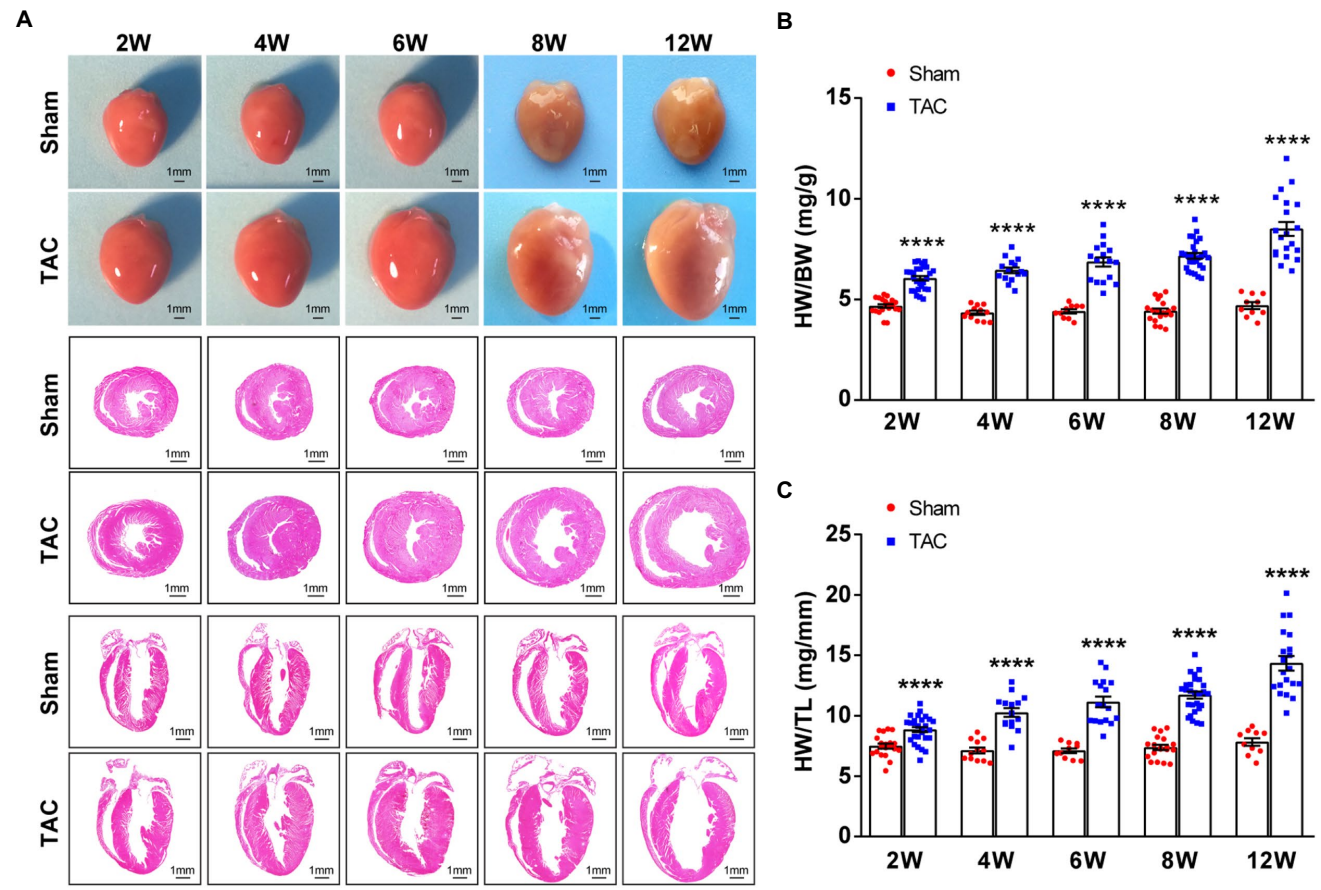
Mouse heart weight and size had a time-series increase after TAC. **(A)** Representative gross heart of mice at various times upon surgery (top), representative images of hematoxylin and eosin (H&E) staining of heart section across the heart’s short axis (transverse section) at various times upon surgery (middle), and representative images of H&E staining of heart section along the heart’s long axis (coronal section) at various times upon surgery (bottom). **(B)** HW/BW ratios at various times upon surgery. **(C)** HW/TL ratios at various times upon surgery. *n* = 10~27. Unpaired *t*-test for two-group comparisons, ^****^p < 0.0001 vs. the corresponding sham.

**Figure 7:**
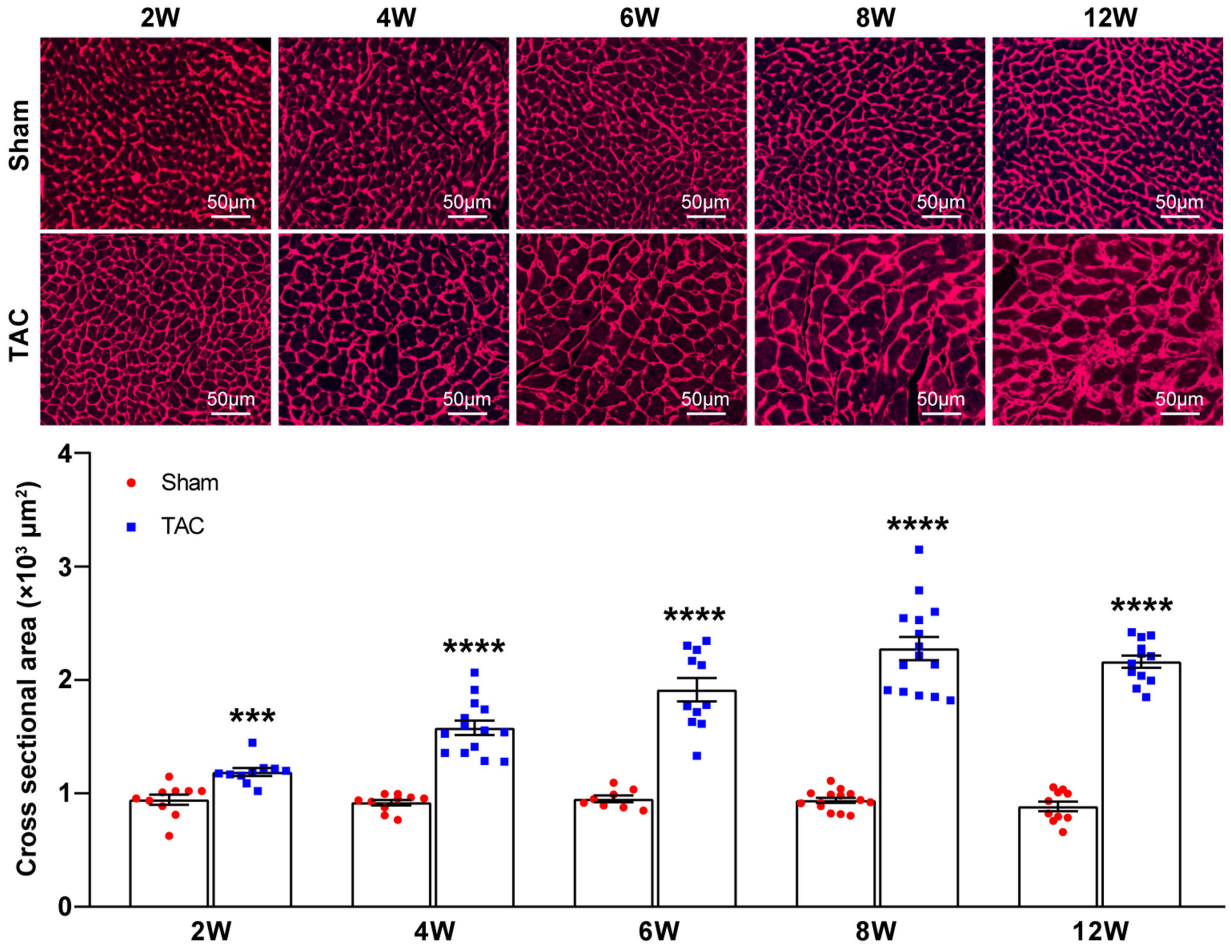
Cardiomyocyte area during TAC-induced time-series cardiac remodeling. Representative images of wheat germ agglutinin staining of heart sections (top). Quantification of cross-sectional areas of cardiomyocytes (bottom), *n* = 8~15. Unpaired *t*-test for two-group comparisons, ^***^p < 0.001, ^****^p < 0.0001 vs. corresponding sham.

In accordance with the echocardiographic data, the heart of the TAC mouse also presented a significantly time-series enlarged and deformed heart (Figure 6A). Accordingly, the heart weight normalized to body weight (HW/BW) and tibia length (HW/TL) were also significantly increased in TAC mice compared to the sham group (Sham 2 W group, *n* = 20; TAC 2 W group, *n* = 27; Sham 4 W group, *n* = 12; TAC 4 W group, *n* = 15; Sham 6 W group, *n* = 10; TAC 6 W group, *n* = 17; Sham 8 W group, *n* = 19; TAC 8 W group, *n* = 27; Sham 12 W group, *n* = 10; TAC 12 W group, *n* = 20; Figures 6B,C). Cardiomyocyte area was also significantly increased in TAC mice compared to the sham group. Further analysis showed that the duration of constriction and cardiomyocyte area of TAC mice gradually increased before 8 weeks, but it had a reduction over 12 weeks (Figure 7).

**Figure 8:**
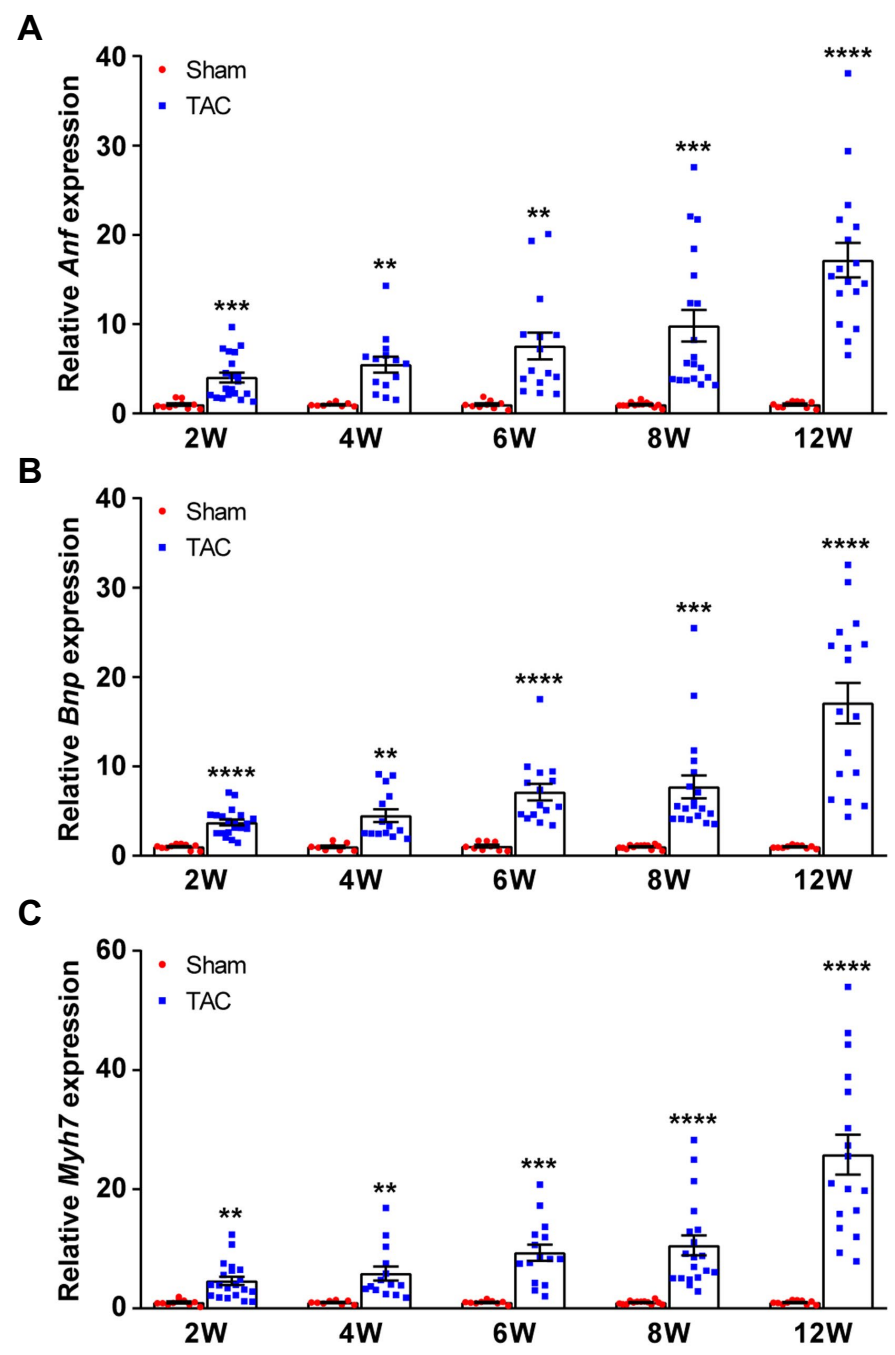
Expression of fetal genes during TAC-induced time-series cardiac remodeling. Expression of the *Anf* **(A)**, *Bnp* **(B)**, and *Myh7* **(C)** in mouse hearts was quantified by qPCR at various times upon surgery. *n* = 8~20. Unpaired *t*-test for two-group comparisons, ^**^p < 0.01, ^***^p < 0.001, ^****^p < 0.0001 vs. the corresponding sham.

The hypertrophic genes atrial natriuretic factor (*Anf*), brain natriuretic peptide (*Bnp*), and β-myosin heavy chain (*Myh7*) were also examined. Results show that the expression of *Anf*, *Bnp*, and *Myh7* had a gradual time-series increase in the TAC mouse heart compared to the sham group (Figures 8A–C). Taken together, these results show that a 25-gauge needle can successfully induce a time-series cardiac remodeling from a compensatory period to a decompensatory period of HF over 8 weeks after TAC.

### A 25-gauge needle TAC induces cardiac fibrosis

**Figure 9:**
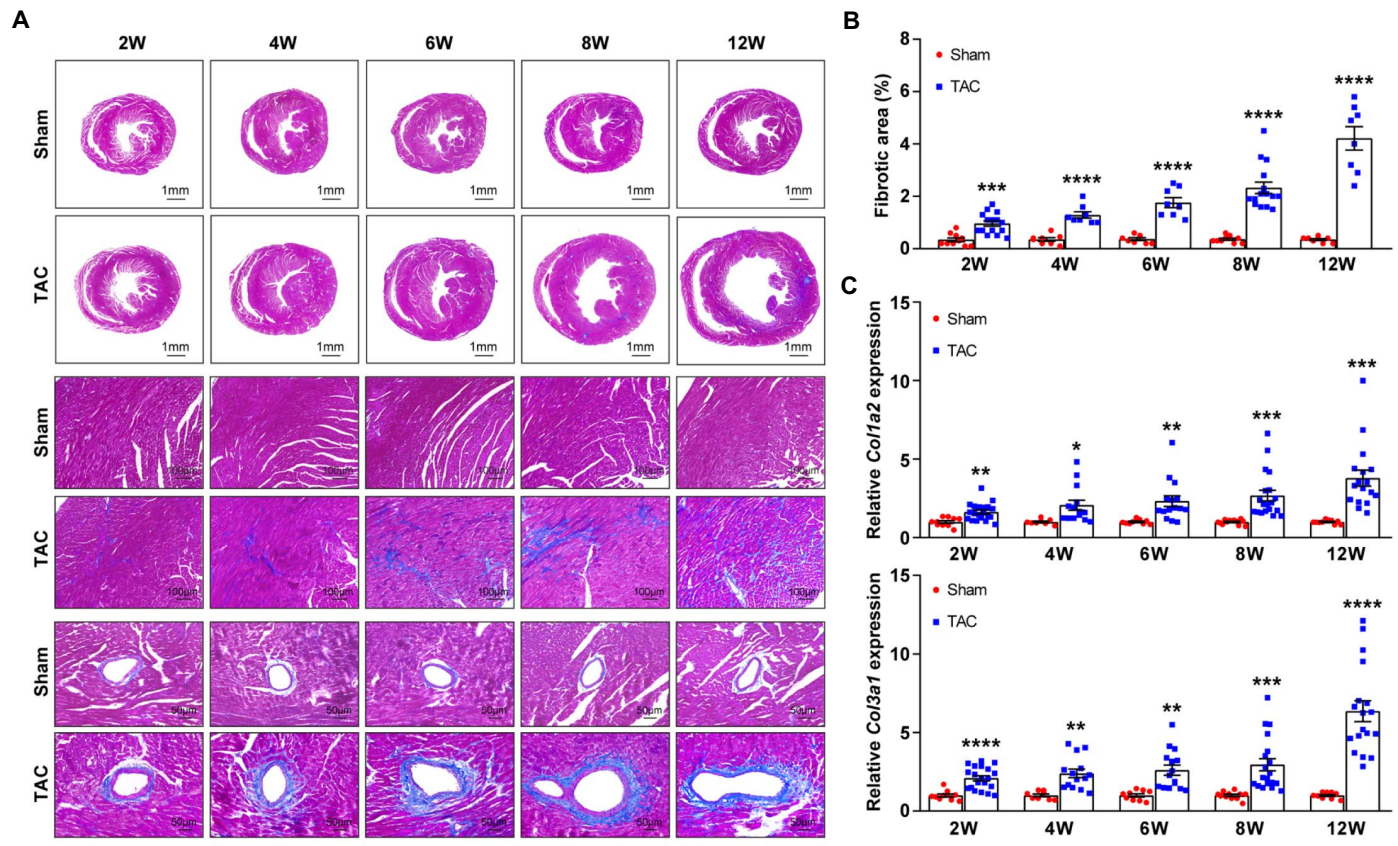
Time-series cardiac fibrosis induced by TAC. **(A)** Representative images of Masson’s trichrome staining of heart sections (top). **(B)** Quantification of the fibrotic area (bottom), *n* = 8~15. **(C)** Expression of *Col1a2* and *Col3a1* in mouse heart was detected by qPCR at various times upon surgery, *n* = 8~20. Unpaired *t*-test for two-group comparisons, ^*^p < 0.05, ^**^p < 0.01, ^***^p < 0.001, ^****^p < 0.0001 vs. corresponding sham.

Cardiac fibrosis plays an important role in the development and progression of HF by causing adverse electrical and mechanical disturbances in diseased hearts (26). Pressure overload-induced cardiac hypertrophy is invariably accompanied by the formation of cardiac fibrosis. Here, the cardiac fibrosis was also analyzed in the mouse heart at different constriction durations by using Masson’s trichrome staining. Analysis showed that compared to the sham group, mice subjected to TAC applied with a 25-gauge needle developed significant cardiac fibrosis (Figures 9A,B). Moreover, cardiac fibrosis showed a time-series gradually increasing trend after TAC. Meanwhile, expression of the fibrosis genes collagen type I (*Col1a2*) and collagen type III (*Col3a1*) was also detected in the mouse heart. The level of these genes corresponded to the degree of cardiac fibrosis area (Figure 9C). Taken together, C57BL/6 J mice subjected to TAC applied with a 25-gauge needle had a time-series increase in cardiac fibrosis that was confirmed by the analysis of Masson’s trichrome staining and fibrosis-related gene expression over 12 weeks after TAC. These results confirm the importance of cardiac fibrosis in the development and progression of HF.

## Discussion

Transverse aortic constriction is a widely used pressure overload model for studying adverse cardiac remodeling and heart failure, and it has been widely used and modified to be minimally invasive since it was first devised to study the mechanisms of cardiac hypertrophy by Rockman in 1991 (7, 12, 14–17, 27, 28). Minimally invasive TAC is a more desirable method for the construction of the aorta arch in mice. It is performed without intubation, ventilation, and entering the chest cavities of mice, involves significantly fewer apparatuses and less damage to mice, leading to a more rapid recovery, and a greater survival rate after surgery. However, the popularity of this minimally invasive TAC method in the study of cardiac remodeling is not high (13). In addition, the degree of constriction resulting from this method is also variable.

The constriction degree of the aorta arch is the most important factor affecting the phenotypes of TAC-induced cardiac remodeling. The constriction diameter of the needles used for TAC varied from 17-gauge to 30-gauge, but a 27-gauge needle has been the most common needle size (>70%) for constriction in TAC study (9, 10, 13, 16, 28). Several studies have compared the resulting phenotypes to different needles, including 25-gauge, 26-gauge, and 27-gauge needles (9, 11). It is reported that the three degrees of tightness TAC models all induced significant, severity-dependent left ventricular hypertrophy and cardiac dysfunction compared to sham mice for 4 weeks. Mice subjected to 27-gauge TAC had the most severe cardiac hypertrophy, cardiac dysfunction, and cardiac fibrosis, and most quickly display features of heart failure compared to 25-gauge and 26-gauge needles. Moreover, it is critical that the suture around the aorta is not too tight during the surgery, because it may lead to a tremendous left ventricular overload and cause a fatal reduction of blood flow to other critical organs, such as the kidneys (11). Taken together, compared to bigger size needles, smaller needles can create a narrower constriction, which results in tremendous left ventricular overload and higher mortality (18). According to the aforementioned reports, a 27-gauge needle is not the best choice for the TAC model, which may be attributable to a too-tight constriction of the aortic arch. However, a 25-gauge needle can cause a much milder constriction of the aorta arch, which is more suitable for the studies of cardiac remodeling in mice, and even studies of more susceptibility to mortality.

Besides the constriction degree of the aorta arch, the duration of aortic constriction is also correlated to the phenotypes of adverse cardiac remodeling (13). However, a time-series of cardiac remodeling phenotypes, induced by TAC applied with a 25-gauge needle in C57BL/6 J mice model, has not been reported. There are cardiac hypertrophy, cardiac dysfunction, cardiac fibrosis, and HF characteristics in TAC-induced cardiac remodeling. Different durations of aortic constriction in mice can induce distinct cardiac remodeling phenotypes and the heart experiences from a compensatory period to an HF period. After TAC surgery, most experiments used a follow-up time of 28 days (16), some followed up by 7, 14, or 56 days (9, 11, 13, 16, 17, 23), and rarely 84 days (20). Moreover, when part of the mice subjected to TAC still showed compensated cardiac hypertrophy, some mice developed HF (29). Therefore, understanding this time-series variability involved in a TAC model is crucial to appropriate study design and interpretation, particularly when investigating the effects of conditional expression of genetic interventions or pharmacologic agents.

Furthermore, C56BL/6 J mice subjected to 25-gauge TAC surgery in studies were reported to be in a compensatory cardiac hypertrophy stage at 4 weeks after TAC, but the specific timeline for the development of heart failure still was not clear (11). In our study, this question has been answered. At 2 weeks after TAC, mice subjected to TAC applied with a 25-gauge needle were in a compensatory period of cardiac remodeling, and the cardiac dysfunction began to appear 4 weeks after surgery. Most of the mice had developed cardiac dysfunction at 6 weeks after TAC. Over 8 weeks, the mice subjected to TAC had developed to the stage of decompensatory HF. Moreover, there was a dilated HF at 12 weeks after TAC. These results are different from that in a study of congestive heart failure (20). Moreover, this method greatly shortens the time of HF induced by pressure overload in C56BL/6 J mice (30).

In this study, the minimally invasive TAC mice model was applied to pressure overload-induced cardiac remodeling and HF in C57BL/6 J mice. Compared to conventional TAC (12), this operation was easier to perform and improved the survival rate. Furthermore, the 2,2,2-tribromoethanol anesthetic and a 25-gauge needle constriction were used to replace the ketamine/xylazine anesthetics and a 27-gauge ligation needle, which were mostly used in reports (9, 13, 16, 17). A 25-gauge needle produced a much milder pressure overload on the left ventricular and had higher survival in this TAC study compared to the effects of a 27-gauge needle (9, 11). Therefore, a 25-gauge needle is much more suitable for studying hypertensive heart diseases. In addition, the time-series of TAC-induced cardiac remodeling phenotypes were observed due to different durations of left ventricular pressure overload by a 25-gauge ligation needle in C57BL/6 J mice. Results showed that the mice subjected to TAC were in the compensatory period of cardiac hypertrophy 2 weeks after the operation, accompanied by the trend of increased cardiac function. Then, the transition from compensatory hypertrophy to early cardiac insufficiency was reflected in the continuous decline of cardiac function within 4~8 weeks. Finally, TAC mice showed an obvious characteristic of heart failure at 8 weeks after TAC and they developed to the stage of dilated heart failure at 12 weeks after the operation. Therefore, in C57BL/6 J mice, TAC applied with 25-gauge needles induced a time-series phenotype of cardiac remodeling with the duration of constriction, and the stage of HF was successfully induced after 8 weeks or later post-TAC. These results are slightly different from previous reports (11, 20).

Despite the homogeneity in experimental design, the TAC model contains a substantial degree of heterogeneity in the outcome measures. Moreover, most studies do not report the mouse number used for each outcome measure (13). This is mainly due to the individual differences among mice in the study and the considerable variability within the same strain (30, 31). This variability within the experiment affects the sample size required to detect the effects of treatment or genetic alterations. Therefore, increasing the experimental sample size is a good way to solve this phenomenon. It is reported that 16%–17% of studies used ≤5 animals in the TAC group, and most articles did not report the number of animals used for each parameter measure, thus, the reproducibility of these results might be of concern (13, 29). In view of the very low number of animals in previous TAC studies, the sample size of each group in this study was increased to adequate numbers (>8) to ensure the reliability of the evaluation parameters, especially the numbers in TAC groups, which is imperative for interventions or pharmacologic agent study in future. Moreover, excepting for histomorphology analysis, the other analysis results in this study, especially survival rate, also collected experimental data from other C57BL/6 J TAC models in our laboratory (these data will not be used in future), which were conducted by the same operator in our team.

In summary, the present study provides a comprehensive analysis of the time-series cardiac remodeling phenotypes induced by different durations of the TAC model applied with a 25-gauge needle in C57BL/6 J mice. Moreover, this TAC method is suitable for high-precision aortic contraction in mice that need high reproducibility and low post-operative mortality. These data have important guiding significance and research value for the research of cardiac remodeling and heart failure in the future. However, there are still some limitations in the current study. The TAC surgery is also affected by the sex, strain, and age of animals, therefore, the data in the present study may not apply to females, other strains, or different age mice.

## Data availability statement

The original contributions presented in the study are included in the article/supplementary material, further inquiries can be directed to the corresponding author.

## Ethics statement

The animal study was reviewed and approved by the Ethics Board of the Capital Medical University.

## Author contributions

XW designed the study, performed the experiments, and wrote the manuscript. XZ, LS, and BY performed the experiments and analyzed the data. JW participated in the mice breeding and management. QX participated in the echocardiography. AQ conceived the study, supervised the study, and wrote the manuscript. All authors contributed to the article and approved the submitted version.

## Funding

This study was supported by the National Natural Science Foundation of China (81800233 and 82070474), the China Postdoctoral Science Foundation (2017M620830), the Beijing Postdoctoral Research Foundation (2018-22-113), and the Key Science and Technology Project of Beijing Municipal Institutions (KZ202010025032).

## Conflict of interest

The authors declare that the research was conducted in the absence of any commercial or financial relationships that could be construed as a potential conflict of interest.

## Publisher’s note

All claims expressed in this article are solely those of the authors and do not necessarily represent those of their affiliated organizations, or those of the publisher, the editors and the reviewers. Any product that may be evaluated in this article, or claim that may be made by its manufacturer, is not guaranteed or endorsed by the publisher.

## References

[1] Roger VL. Epidemiology of heart failure: a contemporary perspective. Circ Res. (2021) 128:1421–34. doi: 10.1161/CIRCRESAHA.121.318172 33983838

[2] Heidenreich PA Bozkurt B Aguilar D Allen LA Byun JJ Colvin MM. 2022 AHA/ACC/HFSA guideline for the Management of Heart Failure: executive summary: a report of the American College of Cardiology/American Heart Association joint committee on clinical practice guidelines. Circulation. (2022) 145:e876–94. doi: 10.1161/CIR.0000000000001062, PMID: 35363500

[3] Ambrosy AP Fonarow GC Butler J Chioncel O Greene SJ Vaduganathan M. The global health and economic burden of hospitalizations for heart failure: lessons learned from hospitalized heart failure registries. J Am Coll Cardiol. (2014) 63:1123–33. doi: 10.1016/j.jacc.2013.11.053 24491689

[4] Frantz S Bauersachs J Ertl G. Post-infarct remodelling: contribution of wound healing and inflammation. Cardiovasc Res. (2009) 81:474–81. doi: 10.1093/cvr/cvn292, PMID: 18977766 PMC2639128

[5] Kannan A Janardhanan R. Hypertension as a risk factor for heart failure. Curr Hypertens Rep. (2014) 16:447. doi: 10.1007/s11906-014-0447-7 24792121

[6] Di Palo KE Barone NJ. Hypertension and heart failure: prevention, targets, and treatment. Heart Fail Clin. (2020) 16:99–106. doi: 10.1016/j.hfc.2019.09.001 31735319

[7] Wang X Ye Y Gong H Wu J Yuan J Wang S. The effects of different angiotensin II type 1 receptor blockers on the regulation of the ACE-AngII-AT1 and ACE2-Ang(1-7)-mas axes in pressure overload-induced cardiac remodeling in male mice. J Mol Cell Cardiol. (2016) 97:180–90. doi: 10.1016/j.yjmcc.2016.05.012, PMID: 27210827

[8] Yu Y Hu Z Li B Wang Z Chen S. Ivabradine improved left ventricular function and pressure overload-induced cardiomyocyte apoptosis in a transverse aortic constriction mouse model. Mol Cell Biochem. (2019) 450:25–34. doi: 10.1007/s11010-018-3369-x, PMID: 29790114

[9] Deng H Ma LL Kong FJ Qiao Z. Distinct phenotypes induced by different degrees of transverse aortic constriction in C57BL/6N mice. Front Cardiovasc Med. (2021) 8:641272. doi: 10.3389/fcvm.2021.641272, PMID: 33969009 PMC8100039

[10] Li H Liu Q Wang S Huang L Huang S Yue Y. A new minimally invasive method of transverse aortic constriction in mice. J Cardiovasc Transl Res. (2022) 15:635–43. doi: 10.1007/s12265-021-10170-4, PMID: 34498212

[11] Richards DA Aronovitz MJ Calamaras TD Tam K Martin GL Liu P. Distinct phenotypes induced by three degrees of transverse aortic constriction in mice. Sci Rep. (2019) 9:5844. doi: 10.1038/s41598-019-42209-7, PMID: 30971724 PMC6458135

[12] Rockman HA Ross RS Harris AN Knowlton KU Steinhelper ME Field LJ. Segregation of atrial-specific and inducible expression of an atrial natriuretic factor transgene in an in vivo murine model of cardiac hypertrophy. Proc Natl Acad Sci U S A. (1991) 88:8277–81. doi: 10.1073/pnas.88.18.8277, PMID: 1832775 PMC52490

[13] Bosch L de Haan JJ Bastemeijer M van der Burg J van der Worp E Wesseling M. The transverse aortic constriction heart failure animal model: a systematic review and meta-analysis. Heart Fail Rev. (2021) 26:1515–24. doi: 10.1007/s10741-020-09960-w, PMID: 32335789 PMC8510918

[14] Eichhorn L Weisheit CK Gestrich C Peukert K Duerr GD Ayub MA. A closed-chest model to induce transverse aortic constriction in mice. J Vis Exp. (2018):134. doi: 10.3791/57397, PMID: 29683463 PMC5933408

[15] Liu B Li A Gao M Qin Y Gong G. Modified protocol for a mouse heart failure model using minimally invasive transverse aortic constriction. STAR Protoc. (2020) 1:100186. doi: 10.1016/j.xpro.2020.100186, PMID: 33377080 PMC7757422

[16] Tavakoli R Nemska S Jamshidi P Gassmann M Frossard N. Technique of minimally invasive transverse aortic constriction in mice for induction of left ventricular hypertrophy. J Vis Exp. (2017):127. doi: 10.3791/56231, PMID: 28994784 PMC5752328

[17] Zaw AM Williams CM Law HK Chow BK. Minimally invasive transverse aortic constriction in mice. J Vis Exp. (2017):121. doi: 10.3791/55293, PMID: 28362400 PMC5409346

[18] Furihata T Kinugawa S Takada S Fukushima A Takahashi M Homma T. The experimental model of transition from compensated cardiac hypertrophy to failure created by transverse aortic constriction in mice. Int J Cardiol Heart Vasc. (2016) 11:24–8. doi: 10.1016/j.ijcha.2016.03.007, PMID: 28616522 PMC5441312

[19] Calamaras TD Baumgartner RA Aronovitz MJ McLaughlin AL Tam K Richards DA. Mixed lineage kinase-3 prevents cardiac dysfunction and structural remodeling with pressure overload. Am J Physiol Heart Circ Physiol. (2019) 316:H145–59. doi: 10.1152/ajpheart.00029.2018, PMID: 30362822 PMC6383356

[20] Tannu S Allocco J Yarde M Wong P Ma X. Experimental model of congestive heart failure induced by transverse aortic constriction in BALB/c mice. J Pharmacol Toxicol Methods. (2020) 106:106935. doi: 10.1016/j.vascn.2020.106935, PMID: 33096237

[21] Barrick CJ Rojas M Schoonhoven R Smyth SS Threadgill DW. Cardiac response to pressure overload in 129S1/SvImJ and C57BL/6J mice: temporal- and background-dependent development of concentric left ventricular hypertrophy. Am J Physiol Heart Circ Physiol. (2007) 292:H2119–30. doi: 10.1152/ajpheart.00816.2006, PMID: 17172276

[22] Wang X Wang HX Li YL Zhang CC Zhou CY Wang L. MicroRNA let-7i negatively regulates cardiac inflammation and fibrosis. Hypertension. (2015) 66:776–85. doi: 10.1161/HYPERTENSIONAHA.115.05548, PMID: 26259595

[23] Wang X Zhu XX Jiao SY Qi D Yu BQ Xie GM. Cardiomyocyte peroxisome proliferator-activated receptor alpha is essential for energy metabolism and extracellular matrix homeostasis during pressure overload-induced cardiac remodeling. Acta Pharmacol Sin. (2022) 43:1231–42. doi: 10.1038/s41401-021-00743-z, PMID: 34376812 PMC9061810

[24] Scherrer-Crosbie M Thibault HB. Echocardiography in translational research: of mice and men. J Am Soc Echocardiogr. (2008) 21:1083–92. doi: 10.1016/j.echo.2008.07.001, PMID: 18723318 PMC2648388

[25] Hartley CJ Reddy AK Madala S Michael LH Entman ML Taffet GE. Doppler estimation of reduced coronary flow reserve in mice with pressure overload cardiac hypertrophy. Ultrasound Med Biol. (2008) 34:892–901. doi: 10.1016/j.ultrasmedbio.2007.11.019, PMID: 18255218 PMC2453594

[26] Schelbert EB Fonarow GC Bonow RO Butler J Gheorghiade M. Therapeutic targets in heart failure: refocusing on the myocardial interstitium. J Am Coll Cardiol. (2014) 63:2188–98. doi: 10.1016/j.jacc.2014.01.068 24657693

[27] Martin TP Robinson E Harvey AP MacDonald M Grieve DJ Paul A. Surgical optimization and characterization of a minimally invasive aortic banding procedure to induce cardiac hypertrophy in mice. Exp Physiol. (2012) 97:822–32. doi: 10.1113/expphysiol.2012.065573, PMID: 22447975

[28] Schnelle M Catibog N Zhang M Nabeebaccus AA Anderson G Richards DA. Echocardiographic evaluation of diastolic function in mouse models of heart disease. J Mol Cell Cardiol. (2018) 114:20–8. doi: 10.1016/j.yjmcc.2017.10.006, PMID: 29055654 PMC5807035

[29] Mohammed SF Storlie JR Oehler EA Bowen LA Korinek J Lam CS. Variable phenotype in murine transverse aortic constriction. Cardiovasc Pathol. (2012) 21:188–98. doi: 10.1016/j.carpath.2011.05.002, PMID: 21764606 PMC3412352

[30] Melleby AO Romaine A Aronsen JM Veras I Zhang L Sjaastad I. A novel method for high precision aortic constriction that allows for generation of specific cardiac phenotypes in mice. Cardiovasc Res. (2018) 114:1680–90. doi: 10.1093/cvr/cvy141, PMID: 29878127

[31] Hermans H Swinnen M Pokreisz P Caluwe E Dymarkowski S Herregods MC. Murine pressure overload models: a 30-MHz look brings a whole new "sound" into data interpretation. J Appl Physiol. (1985) 2014:563–71. doi: 10.1152/japplphysiol.00363.2014 25059236

